# Genome sequencing and secondary metabolism of the postharvest pathogen *Penicillium griseofulvum*

**DOI:** 10.1186/s12864-015-2347-x

**Published:** 2016-01-05

**Authors:** Houda Banani, Marina Marcet-Houben, Ana-Rosa Ballester, Pamela Abbruscato, Luis González-Candelas, Toni Gabaldón, Davide Spadaro

**Affiliations:** DiSAFA - Dept. Agricultural, Forestry and Food Sciences and AGROINNOVA – Centre of Competence for the Innovation in the Agroenvironmental Sector, University of Torino, Largo Paolo Braccini 2, I-10095 Grugliasco, Torino, Italy; Bioinformatics and Genomics Programme. Centre for Genomic Regulation (CRG), Dr. Aiguader 88, 08003 Barcelona, Spain; Universitat Pompeu Fabra (UPF), 08003 Barcelona, Spain; Instituto de Agroquímica y Tecnología de Alimentos (IATA-CSIC), Avda. Agustin Escardino 7, Paterna, Valencia 46980 Spain; Bioeconomy Unit, Parco Tecnologico Padano, via Einstein, 26900 Lodi, Italy; Institució Catalana de Recerca i Estudis Avançats (ICREA), Pg. Lluís Companys 23, 08010 Barcelona, Spain

**Keywords:** *Penicillium griseofulvum*, genome sequencing, secondary metabolites, postharvest disease, blue mold, patulin, roquefortine C, griseofulvin

## Abstract

**Background:**

*Penicillium griseofulvum* is associated in stored apples with blue mould, the most important postharvest disease of pome fruit. This pathogen can simultaneously produce both detrimental and beneficial secondary metabolites (SM). In order to gain insight into SM synthesis in *P. griseofulvum* in vitro and during disease development on apple, we sequenced the genome of *P. griseofulvum* strain PG3 and analysed important SM clusters.

**Results:**

PG3 genome sequence (29.3 Mb) shows that *P. griseofulvum* branched off after the divergence of *P. oxalicum* but before the divergence of *P. chrysogenum*. Genome-wide analysis of *P. griseofulvum* revealed putative gene clusters for patulin, griseofulvin and roquefortine C biosynthesis. Furthermore, we quantified the SM production in vitro and on apples during the course of infection*.* The expression kinetics of key genes of SM produced in infected apple were examined. We found additional SM clusters, including those potentially responsible for the synthesis of penicillin, yanuthone D, cyclopiazonic acid and we predicted a cluster putatively responsible for the synthesis of chanoclavine I.

**Conclusions:**

These findings provide relevant information to understand the molecular basis of SM biosynthesis in *P. griseofulvum,* to allow further research directed to the overexpression or blocking the synthesis of specific SM.

**Electronic supplementary material:**

The online version of this article (doi:10.1186/s12864-015-2347-x) contains supplementary material, which is available to authorized users.

## Background

Blue mould is the most common postharvest disease of cold stored apples and pears [1]. Besides *Penicillium expansum*, the main causal agent of blue mould, other species of *Penicilllium* can cause blue mould on fruit, including *Penicillium chrysogenum, Penicillium crustosum, Penicillium griseofulvum*, and *Penicillium solitum* [2–5]. *Penicillium griseofulvum* Dierckx (syn. *P. patulum* Bain.; *P. urticae* Bain.) can cause blue mould without being associated with *P. expansum*: apples cv Golden Delicious stored in Italy for 6 months showed blue mould and *P. griseofulvum* was the only species isolated from the rots [6]. *P. griseofulvum* is globally distributed and has been isolated also from other fruit species and various environments such as desert soil, cereal grains and animal feed [7].

Classification of *Penicillium* moulds, which comprises over 350 recognized species [8], has evolved since their first description. Most mycologists use primarily morphological criteria, which alone do not allow unambiguous classification. Therefore, classification within the genus *Penicillium* has been revised on the basis of various newly considered criteria, among which the production of specific secondary metabolites (SM) has been considered [9, 10].

SM are structurally heterogeneous low-molecular-mass molecules, and although chemically diverse, they are produced by few core biosynthetic pathways [11, 12]. Some SM are harmful mycotoxins, such as patulin [13]. Others are medically attractive as drugs and are used to efficiently combat human disease, for example penicillin or griseofulvin. Sometimes detrimental and beneficial SM can be produced by the same species, as in *P. griseofulvum* [7, 14], which is known to produce both patulin and griseofulvin. *P. griseofulvum* is also known to be able to synthesize other secondary metabolites, such as roquefortine C, penicillin, cyclopiazonic acid and chanoclavine I [10, 15, 16]. To elucidate the molecular and enzymatic mechanisms underlying the biosynthesis of a specific SM compound, a crucial step is the identification of the biosynthetic genes, which are generally arranged in clusters [11, 17]. So far, apart from penicillin, no gene cluster has been yet identified for the biosynthesis of important secondary metabolites produced by *P. griseofulvum* [10]. Recently, comparative genomics and transcriptomics tools have revolutionized the research on SM gene clusters in fungi, including *Penicillia* [17–20].

Patulin is a toxic fungal compound which produces acute and chronic toxicity, mainly genotoxicity, immunotoxicity, as well as cytotoxicity [13]. Assessment of the health risks due to patulin consumption by humans has led many countries to regulate its amount in food [21]. The responsible biosynthetic gene cluster for patulin was first described in *P. expansum* and *Aspergillus clavatus* [19, 20, 22, 23]. The genetic content of the clusters described in the two species, comprising 15 genes, seemed to be identical, but the gene order greatly varied. In addition a *P. expansum* strain was found to contain the patulin cluster but was unable to synthesize the compound [19]. So far, no information is available about the patulin cluster in *P. griseofulvum*, which is necessary to better understand the mechanisms leading to patulin production in this fungus and to define strategies for patulin reduction in food.

Besides patulin, studies have shown that *P. griseofulvum* is able to produce the mycotoxin roquefortine C [15]. Roquefortine C was first isolated from *Penicillium roqueforti* and since then it has been reported from 25 different *Penicillium* species [10], that grow on different hosts and matrices, such as contaminated beer, feed grain, and onions [24, 25]. Roquefortine C contamination of food and feedstuff is of relevance because of the well-known neurotoxicity of this mycotoxin [26]. In 2011, the roquefortine C biosynthesis pathway was discovered for the first time in *P. chrysogenum* [24]. Later on, this pathway was refined and the exact roquefortine/meleagrin (R/M) biosynthetic gene cluster with the corresponding pathway was elucidated [27, 28]. Although the cluster is composed of seven genes, only three of them are needed for roquefortine production in *P. chrysogenum* (*roqA*, *roqD*, *roqR*) [27, 28]. Recently, Kosalková and collaborators [29] reported the roquefortine gene cluster in *P. roqueforti*, which is shorter than the *P. chrysogenum* R/M seven-gene cluster. This cluster leads to the synthesis of roquefortine C rather than meleagrin as a final product. Considering that *P. griseofulvum* is frequently isolated from apple, corn, wheat, barley, flour, walnuts, and from meat products [6, 30], it could be a potential source of roquefortine C in food. Therefore, it is of great interest to study the genes encoding the roquefortine C production in this fungal species.

*P. griseofulvum* is known to produce also useful SM, including griseofulvin [7, 10]. Besides its recognized antifungal properties against a wide variety of plant pathogens [31], griseofulvin has been used for many years in medical and veterinary applications [32]. Recently, its potential use in cancer chemotherapy has been recognized due to its specific antimitotic and antiproliferative action toward cancer cells [33–35]. Interestingly, griseofulvin has also shown to suppress the replication of the hepatitis C virus *in vitro* [36]. The biosynthetic pathway of griseofulvin has been extensively studied using isotopic incorporation [37–39]. The biosynthetic gene cluster consisting of 13 putative genes has been reported in *Penicillium aethiopicum* [40–42], but the genes forming the griseofulvin cluster in *P. griseofulvum* are still unknown.

Here we report the complete genome sequence of *P. griseofulvum* strain PG3 and describe some important SM clusters present in the studied strain, the expression of their key genes, as well as the analysis of their SM production both *in vitro* and *in vivo*. This resource will allow further insight into SM synthesis in *P. griseofulvum.*

## Results and discussion

### Morphological and molecular identification

The strain PG3 was identified by morphological and microscopic observation. Colonies grown on PDA at 24 °C for 7 days attained 28 mm diameter, with a rich conidiation (Additional file 1: Figure S1A). Colour is grey-green, while reverse is orange-brown (Additional file 1: Figure S1B). PG3 morphological and microscope description corresponded to *P. griseofulvum* species description [43].

Sequencing of the ribosomal regions ITS1–5.8S–ITS2 with universal primers ITS-1 and ITS-4 confirmed the morphological results (GenBank accession number KJ467353.1; amplicon size: 559 bp), showing 99 % (544/548) identity with the sequences of *P. griseofulvum* (accession numbers GU566212.1 and GU566224.1)*.*

### Genome sequencing and comparative genomics

The genome assembly of *P. griseofulvum* strain PG3 is composed of 363 contigs, 14 of them were larger than 100 kb (Table 1). The estimated genome size was 29.3 Mb. Gene annotation showed that 9,631 putative proteins were encoded in the genome. The comparison of PG3 beta-tubulin and calmodulin sequences with several *Penicillium* species present in Genbank confirmed the taxonomic classification assigned to the species *P. griseofulvum* (100 % identity with JF909942.1 and 99.75 % identity with JX996966.1, respectively; Additional file 1: Figure S2).

**Table 1:** Statistics about *P. griseofulvum* PG3 genome assembly.

	***Penicillium griseofulvum*** **PG3**
Genome size	29.3 Mb
Number of contigs	363
Number of contigs > 100 kb	14
N50	2.8 Mb
Number of Ns	322
GC content	0.47
Number of predicted proteins	9631
Average protein length	521 aa

**Fig. 1:**
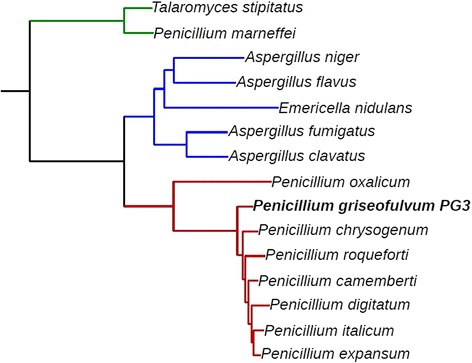
Maximum likelihood species tree of PG3 and the other fourteen studied species. The species tree is derived from gene concatenation of 2,134 single-copy genes present in the newly sequenced genome PG3, in the other sequenced *Penicillium* genomes (*P. chrysogenum, P. oxalicum, P. roqueforti, P. camemberti, P. expansum, P. digitatum* and *P. italicum*), and in members of the *Aspergillus* and *Talaromyces* clades

We compared the genome of *P. griseofulvum* with the genomes of 14 other fully-sequenced *Penicillium* and *Aspergillus* species. To determine the phylogenetic position of *P. griseofulvum* in relation with the other sequenced species, we reconstructed a species tree based on the concatenation of 2,134 genes that were found to be single copy in all considered species. Our results show that *P. griseofulvum* branched off after the divergence of *Penicillium oxalicum* but before the divergence of *P. chrysogenum* (Fig. 1).

We used SMURF [44] in order to predict all the possible SM clusters encoded in *P. griseofulvum.* SMURF was able to identify 47 SM gene clusters with 66 signature proteins, which puts it on level with *P. expansum* in terms of capacity to synthesize SM compounds and above any other sequenced *Penicillium* (Additional file 1: Table S1) [19]. Among the signature proteins there were 14 NRPS, 26 PKS, 3 NRPS-PKS hybrids and 6 DMAT proteins. The remaining proteins being similar to NRPS or PKS proteins. These numbers are again similar to the ones found in *P. expansum* with the exception that *P. expansum* has a double amount of NRPS-PKS hybrid proteins and half the amount of the DMAT proteins than *P. griseofulvum*.

### Genome-wide analysis of P. griseofulvum PG3 genes revealed putative gene clusters for patulin, griseofulvin and roquefortine C biosynthesis

The presence of known SM clusters in PG3 was analysed by searching for homologs of 122 known gene clusters taken from the bibliography and 37 additional clusters downloaded from MIBiG (http://mibig.secondarymetabolites.org/) [19, 45] (see Experimental procedures). We were able to detect the presence of gene clusters putatively responsible for the synthesis of patulin, roquefortine C / meleagrin, griseofulvin, penicillin, cyclopiazonic acid, yanuthone D and chanoclavine I (Additional file 1: Table S2). . Besides the bioinformatic analysis of these gene clusters, we quantified the production of patulin, griseofulvin and roquefortine C by *P. griseofulvum* PG3 which are very important either for their beneficial medical and veterinary applications or because of their toxicity and the risk for human safety*.* The quantification was done in vitro and on apples. Furthermore, the SM detected during apple infection were closely examined by studying the expression kinetics of their key genes under controlled conditions.

### Patulin production by *P. griseofulvum*

**Fig. 2:**
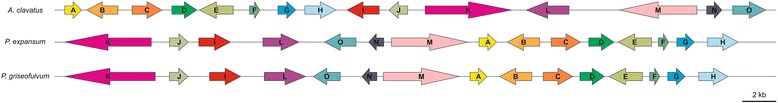
Comparison of patulin gene cluster in PG3 and in phylogenetically close patulin producing species. The species used for comparison are *P. expansum* and *A. clavatus* which have a complete patulin gene cluster [19, 20, 22, 23]. Each gene is indicated by an arrow and named according to its position in the original cluster described in *A. clavatus* [22]

The patulin gene cluster found in *P. griseofulvum* PG3 is formed by 15 genes (Additional file 1: Table S2), positioned in the same gene order as the one found in *P. expansum* [19, 20, 23] (Fig. 2).

The presence of a complete patulin cluster in PG3 is congruent with the results obtained by Ballester and collaborators (2015) [19], who predicted that the presence of this cluster is likely an ancestral trait in *Penicillium* spp., predating the divergence of *P. roqueforti*. Interestingly, the similarities in gene order found between the patulin gene cluster in *P. expansum* and *P. griseofulvum* indicate that the changes in gene order observed between the cluster in *Aspergillus clavatus* and the cluster in *P. griseofulvum* and *P. expansum* happened before the two *Penicillium* species diverged.

**Fig. 3:**
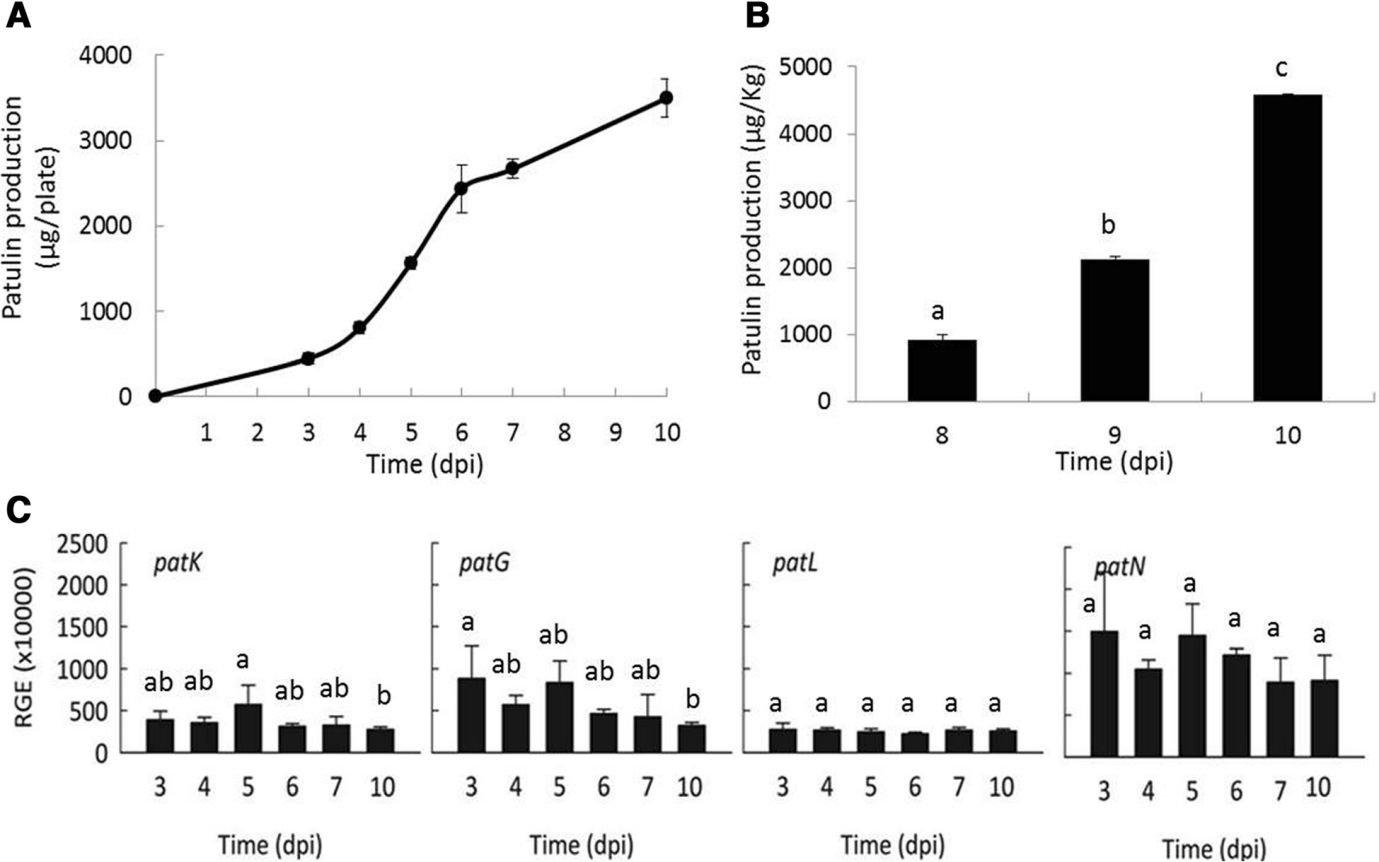
Patulin production and relative expression of key patulin genes in *P. griseofulvum* PG3. **a** Patulin production *in vitro*. **b** Patulin production in apple. **c**. Relative gene expression (RGE) of *patK*, *patG*, *patL* and *patN* in PG3. The gene expression is relative to the geometric mean of the four *P. griseofulvum* reference genes: the ribosomal 28S RNA, the 37S ribosomal protein (PGRI_092740), the beta-tubulin (PGRI_052690) and the histone H3 (PGRI_044770). Spore suspensions of strains were inoculated on the centre of 55 mm PDA plates and incubated at 24 °C in the dark for up to 10 days post inoculation (dpi). Error bars indicate standard deviations of three biological replicates. Different letters above the columns indicate a significant difference determined by Tukey's Multiple Comparison Test (p < 0.05)

Patulin production in *P. griseofulvum* PG3 is considerable (446.86 μg/plate) after 3 days of incubation in vitro and increased significantly to reach 3,498.71 μg/plate at day 10 (Fig. 3A). Interestingly, these concentrations are in the same range as the patulin production found in *P. expansum* strain PEX1 [19], although PG3 exhibited distinct differences in colony morphology and slower growth kinetics compared with PEX1 (Additional file 1: Figure S1).

We further investigated the ability of PG3 to produce patulin in artificially contaminated apples. At 8 dpi, the patulin concentration produced by PG3 was about 914.69 μg patulin/kg of fruit, then it increased as a function of time to reach 4,578.30 μg patulin/kg at 10 dpi (Fig. 3B). The values of patulin produced by the *P. griseofulvum* strain highly exceeded recommended maximum acceptable limits set by the European Union and are similar to those reported by Welke and collaborators (2011) [5].

In addition, we investigated the expression pattern of four genes from the patulin biosynthetic cluster: *patK* (encoding a 6-methylsalicylic acid synthase, the first enzyme in the pathway), *patG* (encoding a 6-methylsalicylic acid decarboxylase, the second enzyme), *patN* (encoding an isoepoxydon dehydrogenase, one of the latest steps in the pathway), and *patL*, which encodes the pathway specific transcription factor (Fig. 3C). The expression profiles were very similar for the four genes and clearly different from those observed in their *P. expansum* orthologous [19]. In PG3, the gene expression levels remained relatively constant or slightly decreased at 10 dpi but the differences were not statistically significant.. In contrast, the expression of *patK*, *patN,* and *patL* in *P. expansum* PEX1 increased over time, reaching a maximum at the latest sampled time, with a 20 to 35 fold induction at 7 dpi with respect to 3 dpi [19]. The difference in gene expression profiles between the two *Penicillium* species could be probably related to the cluster regulatory mechanisms since *PatL* expression barely changed over time in PG3, but it increased in *P. expansum* leading probably to an increased expression of the whole cluster. From these results it is apparent that these two species have developed different mechanisms for regulating patulin production.

### A partial cluster of roquefortine C and meleagrin is responsible for the production of roquefortine C in *P. griseofulvum* PG3.

**Fig. 4:**
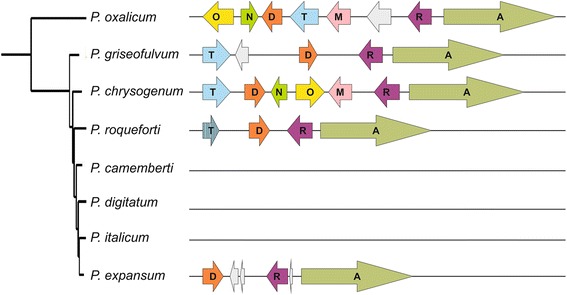
Comparison of the roquefortine C cluster genes in PG3 and in other Penicillium species. The species in which the roquefortine C cluster has been described are *P. chrysogenum* and *P. roqueforti* [27–29]. Each gene is named according to its position in the original cluster described in *P. chrysogenum* [27, 28]*.* Additional genes within the cluster are marked with a grey arrow, and stripped arrow represents a pseudogene

In addition to the patulin gene cluster, we detected the roquefortine C / meleagrin (R/M) gene cluster which is only partially conserved (Fig. 4; Additional file 1: Table S4). It was established that a single cluster of coregulated genes encodes the biosynthesis of both mycotoxins roquefortine C and meleagrin in *P. chrysogenum,* in which this cluster has been well characterized in the last decade [24, 27, 28].

When compared with the *P. chrysogenum* R/M gene cluster, *P. griseofulvum* PG3 contains only four genes *roq*A, *roqR*, *roqD* and *roqT*, encoding roquefortine dipeptide synthetase, roquefortine D dehydrogenase, roquefortine prenyltransferase, and MFS transporter respectively [27, 28], and an additional gene present between *roqD* and *roqT*. The order and orientation of these genes are identical to the *P. chrysogenum* R/M gene cluster.

The three orthologous genes of *roqO*, *roqN,* and *roqM* that are absent in PG3 strain are involved in the late reaction steps of the meleagrin pathway and encode a flavin-dependent N-hydroxylase, a scaffold reorganizing oxygenase and a methyltransferase respectively [27, 28].

Interestingly, Kosalková and collaborators [29] reported that three strains of *P. roqueforti* also contained the R/M core cluster but lacked the genes involved in the conversion of roquefortine C into meleagrin, leading to the synthesis of roquefortine C rather than meleagrin. *P. roqueforti* contains *roqA*, *roqR*, *roqD* and a non-functional pseudogene *roqT* (Fig. 4). However *roqO, roqM* and *roqN* were absent in the *P. roqueforti* strain used in our analysis, whereas they are present in *P. oxalicum,* which is known to produce roquefortine as an intermediate in the biosynthesis of oxaline [29, 46]. We investigated the R/M gene cluster in a group of *Penicillium* genomes and the complete cluster was found only in *P. oxalicum* and *P. chrysogenum* (Fig. 4). *P. oxalicum* presents an inversion of several genes when compared to the cluster of *P. chrysogenum* and has an additional gene located between *roqM* and *roqR*. In *Penicillium digitatum*, *Penicillium italicum,* and *Penicillium camemberti* the R/M gene cluster was lacking, while *P. roqueforti*, *P. expansum,* and *P. griseofulvum* had at least the genes (*roqD, roqR, roqA*) needed to synthesize roquefortine C. The latter species had their remaining genes located in the same order found in *P. chrysogenum* indicating that the inversion happened either specifically in *P. oxalicum* or after its divergence from the other sequenced *Penicillium* species.

In order to confirm that *P. griseofulvum* PG3 was indeed able to synthesize roquefortine C, we studied its production *in vitro* and in artificially infected apples.

**Fig. 5:**
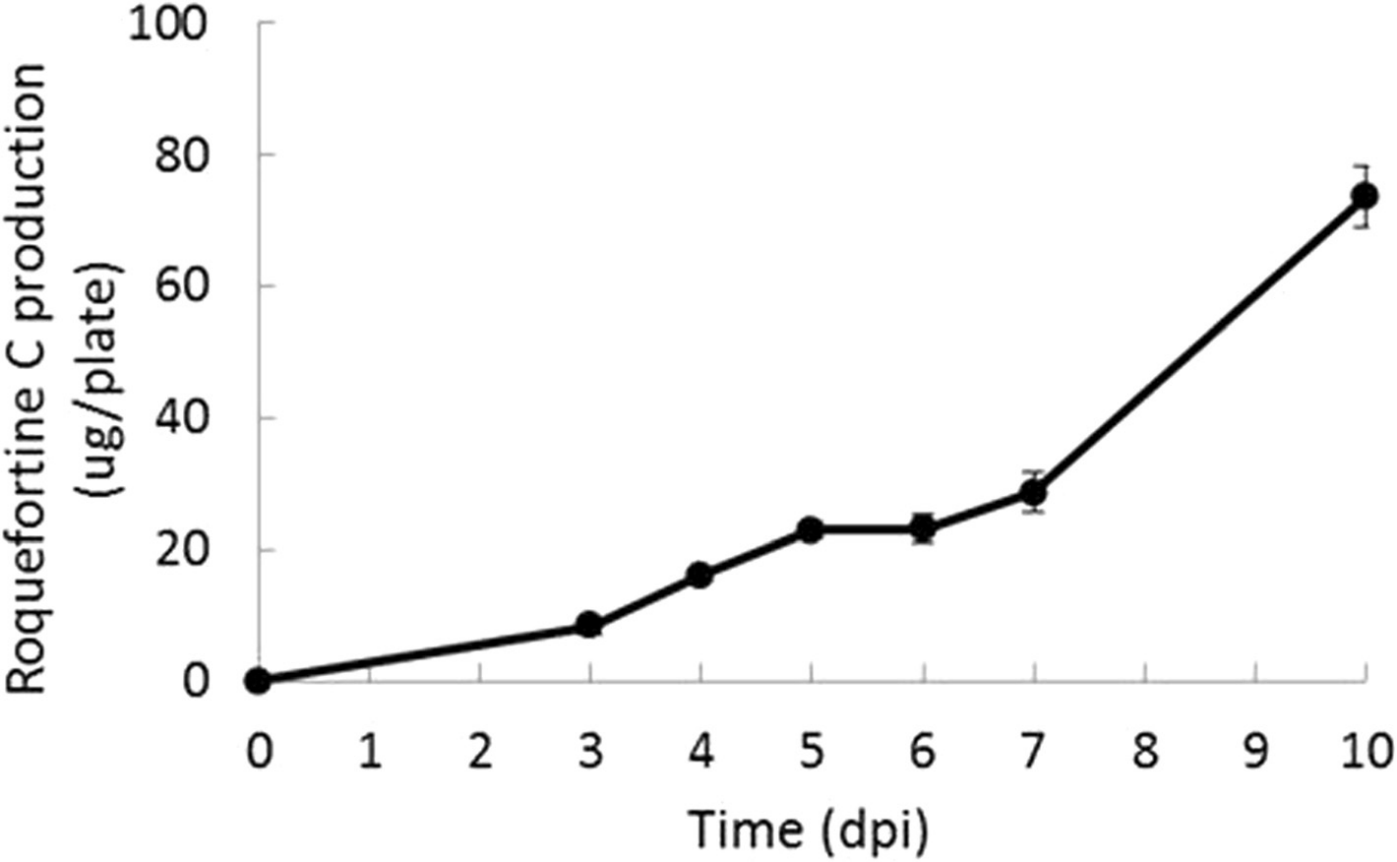
Roquefortine C production by *P. griseofulvum* PG3 *in vitro*

The results showed that PG3 exclusively produced roquefortine C *in vitro*, reaching an amount of 73.65 μg/plate after 10 days of incubation in PDA plates (Fig. 5). As expected, we did not detect the production of meleagrin by PG3.

Naturally occurring roquefortine C has been already detected in apples still on the tree and in windfall apples, however, it has not yet been reported in rotted pome fruits [47, 48].

Interestingly, no roquefortine C production by PG3 was detected in apples during the 10 days of artificial infection, which could be explained by the fact that maybe the apple rot conditions are not favourable for roquefortine C production.

Griseofulvin production by *P. griseofulvum*

As mentioned before, *P. griseofulvum* is known to produce griseofulvin. The gene cluster responsible for the synthesis of this compound was originally described in *P. aethiopicum* [42]. The cluster in *P. aethiopicum* is formed by a polyketide synthase (*gsfA*), three O-methylation proteins (*gsfB*, *gsfC*, and *gsfD*), a chlorination (*gsfI*), a grisan ring formation (*gsfF*), an alkene reduction (*gsfE*), a putative isochorismatase (gsfH), a putative oxidoreductase (gsfK), and an efflux pump (*gsfJ*) [40, 41]. In addition, the griseofulvin cluster in *P. aethiopicum* contained two transcription factors *gsfR1* and *gsfR2*, but it is unclear if both or either of them is involved in the regulation of the cluster [42].

**Fig. 6:**
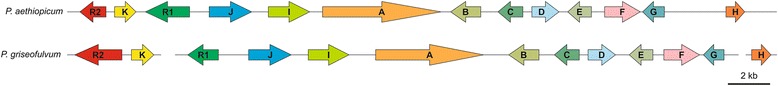
Schematic representation of the griseofulvin gene cluster in *P. aethiopicum* and PG3. Each gene is named according to its position in the original cluster described in *P. aethiopicum* [40–42]

Interestingly, when we compared the griseofulvin gene cluster of *P. aethiopicum* [40–42] with the one found in PG3 (Fig. 6; Additional file 1: Table S5), we found that three genes were not located within the cluster in PG3: *gsfH*, *gsfK* and the transcription factor *gsfR2. gsfH* and *gsfK* are not required for griseofulvin production so their absence from within the cluster is not surprising. The absence of gsfR2 could have an effect on the expression conditions of the cluster but there is an homolog of this protein in another region in the genome therefore we cannot discard the option that it is still playing a function in the regulation of griseofulvin cluster.

A previous study reported that apple medium is one of the best media for griseofulvin and patulin production by *P. griseofulvum* [16], but to our knowledge, no studies have yet explored griseofulvin production by *P. griseofulvum* during apple infection. This analysis could help to identify the suitable conditions for the production of such an important SM in the environment/orchard.

**Fig. 7:**
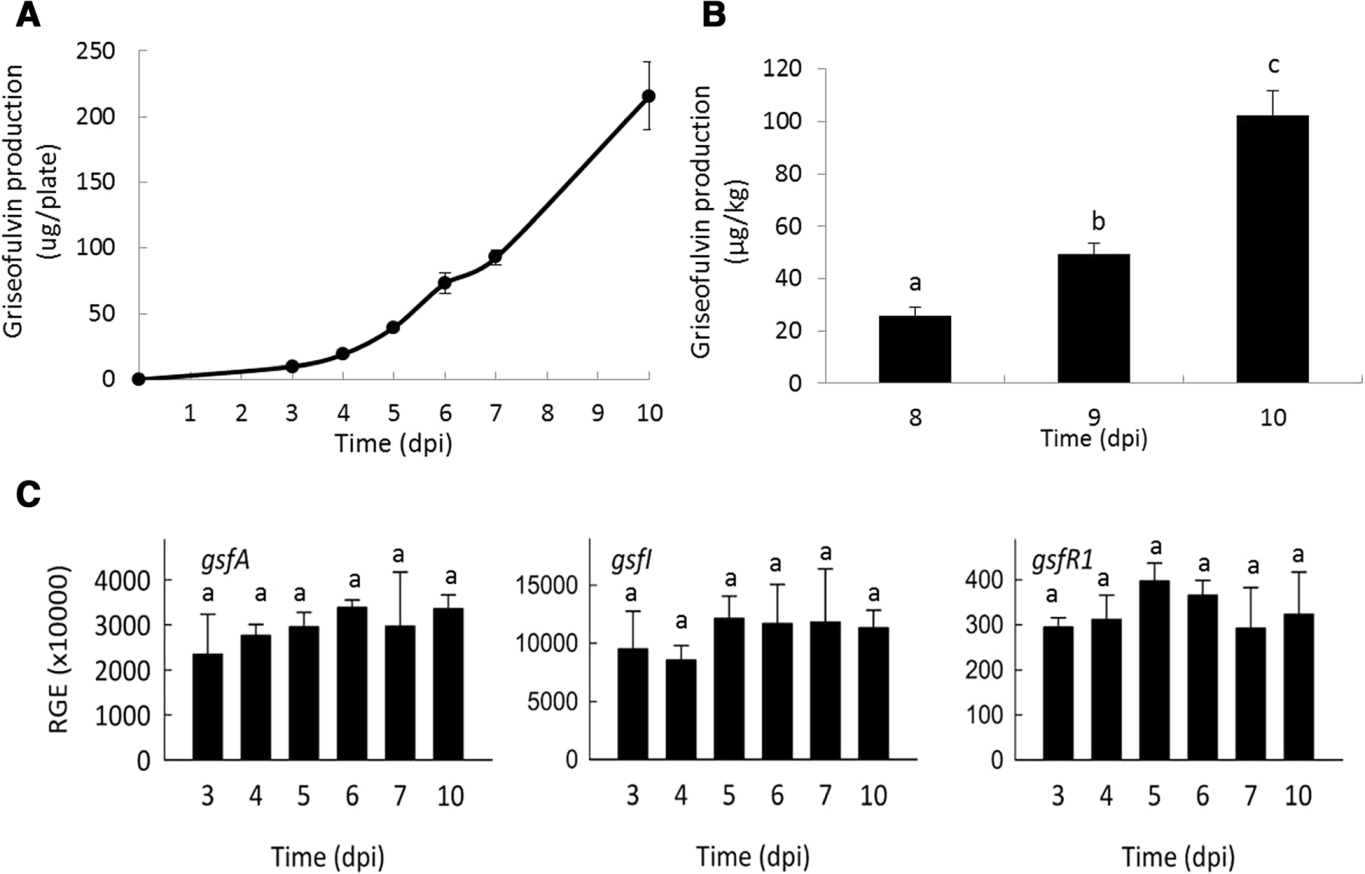
Griseofulvin production and relative expression of key griseofulvin genes in *P. griseofulvum* PG3. **a** Griseofulvin production *in vitro*. **b** Griseofulvin production in apple. **c** Relative gene expression of *gsfA*, *gsfI* and *gsfR1* in *P. griseofulvum* PG3. The gene expression is relative to the geometric mean of the three *P. griseofulvum* reference genes: the ribosomal 28S RNA, the 37S ribosomal protein (PGRI_092740), the tubulin (PGRI_052690) and the histone H3 (PGRI_044770). Error bars indicate standard deviations of three biological replicates. Different letters above the columns indicate a significant difference determined by Tukey's Multiple Comparison Test (p < 0.05)

PG3 produced significant levels of griseofulvin both in vitro and on apples, in increasing amount over the time to reach, at 10 dpi, about 215 μg per plate in vitro and 102.46 μg per kg of apples (Fig. 7 A-B).

The production of griseofulvin by the newly sequenced *P. griseofulvum* PG3 strain indicates that the lack of *gsfR2* within the gene cluster is apparently not affecting the synthesis of griseofulvin under the tested conditions. However we do not know whether or not this gene is nevertheless involved in the synthesis of this compound.

We further analysed the expression of three genes from the griseofulvin gene cluster: *gsfR1*, which codes for a transcription factor, *gsfI*, coding for a halogenase that catalyses the last step in the synthesis of griseofulvin, and *gsfA*, which encodes the polyketide synthase. The three analysed *gsf* genes showed a constitutive expression under the assayed conditions (Fig. 7C). Interestingly, the normalized expression levels of the genes coding for the two structural proteins (*gsfA* and *gsfI*) are much higher than those from the patulin biosynthetic cluster.

### Bioinformatic prediction of additional secondary metabolite clusters

*P. griseofulvum* is known to be able to synthesize penicillin and cyclopiazonic acid [49] but the genes involved in the production of these compounds are still unknown.

**Fig. 8:**
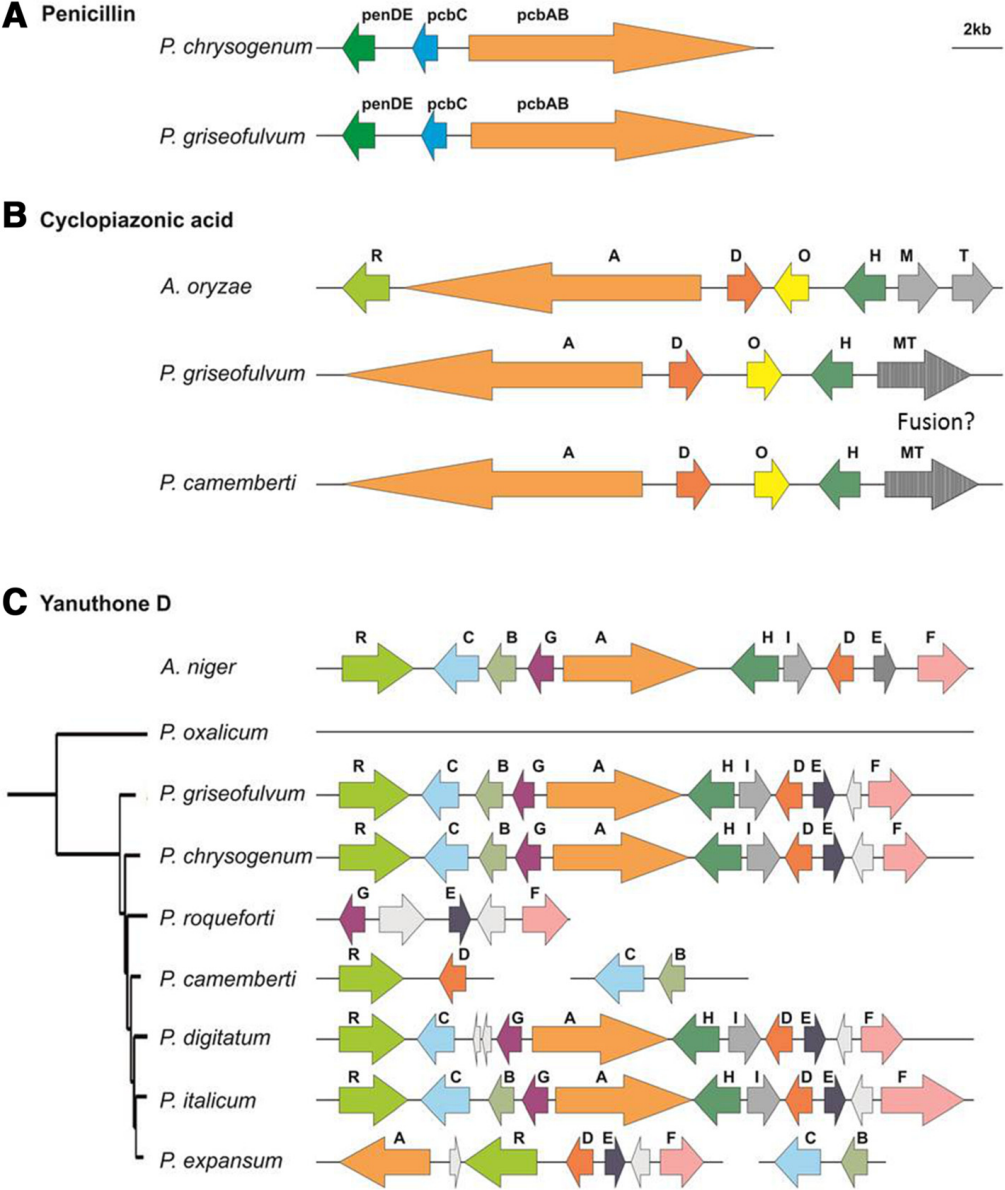
Schematic representation of additional gene clusters in *P. griseofulvum* PG3*.* (A): penicillin, (B): cyclopiazonic acid, (C): yanuthone D. Each gene is named according to its position in the original cluster described in literature.

Therefore we search for additional clusters within PG3 genome to identify putative genes encoding for these SM (Fig. 8A-B; Additional file 1: Tables S6-S7). The gene cluster for penicillin synthesis was found and is homologous to the one found in *P. chrysogenum* (Fig. 8A). The cluster for cyclopiazonic acid synthesis was also found and is homologous to the one described in *Aspergillus oryzae*. The only particularity is that it lacked a regulatory protein, which was also absent in different strains of *A.oryzae* (Fig. 8B)*.* Oddly, in *P. griseofulvum* the genes *cpaT* and *cpaM* are predicted as fused into a single protein, this is possibly due to a frame shift towards the end of *cpaT* which provokes the stop codon misreading. Gene fusion is not uncommon since it has been observed previously in other biosynthetic pathways such as the mycophenolic pathway [50]. The cyclopiazonic gene cluster is only conserved in *P. camemberti* of all the other *Penicillium* species considered and this species shows the same gene fusion.

In addition to the described clusters, we found an additional gene cluster responsible for the synthesis of the antibiotic yanuthone D (Fig. 8C; Additional file 1: Table S8) which was described in *Aspergillus niger* [50]. The cluster is complete and has the same gene order as in *A. niger*, the only peculiarity is that it encodes an additional gene of unknown function. This gene cluster is conserved in other *Penicillium* species, such as *P. chrysogenum* and *P. italicum*, while it was almost completely lost in *P. roqueforti*, *P. camemberti,* and *P. oxalicum* and shows some signs of degradation in *P. expansum* and *P. digitatum*. All *Penicillium* genomes having the cluster contain this additional gene suggesting that it was acquired at the base of *Penicillium* speciation or lost specifically in *A. niger*.

*P. griseofulvum* is also known to synthesize other compounds such as chanoclavine I, cyclopiamide, dehydrofulvic acid, mycelinamide, and phenylpyropene [15]. There is little information about most of these clusters making it difficult to predict which clusters could be able to synthesize these compounds. Despite this, we were able to locate a group of genes that is likely responsible for the synthesis of chanoclavine I. This compound is well known as an intermediate needed to synthesize ergot alkaloids [51]. Several genes have been related to its synthesis in different species such as in *A. japonicus*, *A. fumigatus* and *A. nidulans* [51–53]: a dimethylallyl tryptophan synthase, a methyltransferase, a catalase, and a chanoclavine-I synthase oxidoreductase. We found a group of seven genes clustering together, four of these genes encoded homologs for genes involved in the synthesis of ergot alkaloids in *Epichloe* species. These genes were dimethylallyl tryptophan synthase (*dmaW*), oxidoreductase (*easE*), catalase (*easC*) and phytanoyl-CoA dioxygenase (*easH*). The first three genes are known to synthesize the first steps of the ergot alkaloid pathway and resulting in chanoclavine I (Additional file 1: Table S9). A fourth gene is missing in order to have the complete pathway, a methyltransferase (*easF*). Among the remaining three genes found within the cluster, one of them encoded a methyltransferase that is not homologous to *easF*. It is possible that this gene was transferred at the base of pezizomycotina by *Streptomyces* species as there are only homologs of these gene in *P. griseofulvum*, *P. expansum*, the Dothideomycetes *Cochliobolus heterostrophus* and the Sordariomycetes *Metarhizium anisopliae*. The phylogenetic tree is consistent with this assessment, as it places these small group of fungi as sister group of *Streptomyces* spp..

The presence of three additional genes that are not known to be involved in the synthesis of chanoclavine I opens the door to the possibility that chanoclavine I is, also in *P. griseofulvum*, only an intermediate and that the final compound synthesized by the specific gene cluster is a derivative of chanoclavine I. This gene cluster is conserved also in *P. expansum*, but this species is not known to synthesize chanoclavine I reinforcing the hypothesis that chanoclavine I is not the end product of this cluster.

## Conclusions

The genome of the postharvest pathogen *P. griseofulvum* allowed the identification of a complete putative gene clusters for patulin, griseofulvin and roquefortine C biosynthesis. In addition, we found additional SM clusters, including those potentially responsible for the synthesis of penicillin, yanuthone D, cyclopiazonic acid, and predicted a cluster involved in the synthesis of chanoclavine I. These findings provide relevant information to understand the molecular basis of SM biosynthesis in *P. griseofulvum.* Future studies should consider the role of SM biosynthesis in *P. griseofulvum* in the pathogenetic process of colonization of apple and other fruit species. It is also necessary to better understand the mechanisms leading to patulin production in this fungus to help defining strategies for patulin reduction to avoid consumer health threats and increase food safety. This resource will also allow further research directed to the overexpression or blocking of specific SM synthesis, to assess its potential in terms of biotechnological applications for beneficial SM, such as griseofulvin.

## Methods

### Microorganism and morphological identification

*Penicillium griseofulvum* Dierckx (syn: *P. urticae* Bainier) strain PG3 (deposited at Centraalbureau voor Schimmelcultures, with Accession number CBS 140421) was obtained from rotten apples harvested in Piedmont, Northern Italy. The strain was stored in tubes with potato dextrose agar (PDA; Merck, Darmstadt, Germany) and 50 mg/L streptomycin (Merck) at 4 °C.

The *P. griseofulvum* PG3 isolate was identified based on morphology, microscope observation and DNA sequencing. PG3 was grown on PDA at 24 °C for 10 days, then the colony morphology was observed and the diameter was measured, followed by microscope observation of cell shape and size. For molecular identification, fungal mycelium was scratched with a sterile blade and used for DNA extraction.

### Extraction of genomic DNA

Total DNA was extracted from the strain PG3 as previously described by Ballester and collaborators (2015) [19]. Briefly, spore suspensions of *P. griseofulvum* PG3 (5 × 10^5^ con/mL) were inoculated in 500 mL Potato Dextrose Broth (PDB, Difco) and incubated on a rotary shaker (180 rpm) at 24 °C for 4 days. Fungal mass was filtered from the culture, frozen in liquid nitrogen and DNA was extracted from 1 g frozen mycelium. DNA concentration and purity were checked by a spectrophotometer (Nanodrop 2000, Thermo Scientific, Wilmington, USA), and the DNA integrity was analysed by agarose gel electrophoresis (data not shown).

### Molecular identification

For molecular identification, DNA was PCR amplified using universal primers ITS1 and ITS4 [54]. The PCR program followed was: 95 °C, 3 min; 34 cycles: 94 °C, 15 s; 55 °C, 45 s; 72 °C, 55 s; and a final elongation step at 72 °C for 7 min. PCR amplicons were cloned into the PCR4 TOPO vector (Invitrogen) and sequenced by Genome Express (Padova, Italy) using an ABI PRISM 3730XL DNA Sequencer. ITS sequences were edited with DNA Baser (DNA Baser Sequence Assembler v4.16.0, www.DnaBaser.com) based on the chromatogram and compared to those existing in the NCBI database (www.ncbi.nlm.nih.gov) using BLAST algorithm (E = 0).

### Genome sequencing

The genome of *P. griseofulvum* PG3 was sequenced at the Genomics Platform of the Parco Tecnologico Padano using the Illumina MiSeq technology. Two paired-end and one mate pair libraries were prepared using Nextera XT DNA Sample Preparation Kit and Nextera Mate Pair Sample Preparation Kit, respectively, following the manufacturer’s instructions. Two paired-end (PE) libraries were prepared: PE1 with overlapping paired-end reads and PE2 with non-overlapping paired-end reads. Libraries were purified by AMPure XP beads and normalized to ensure equal library representation in the pools. Equal volumes of libraries were diluted in the hybridization buffer, heat denatured and sequenced. Standard phi X control library (Illumina) was spiked into the denatured HCT 116 library. The libraries and phi X mixture were finally loaded into a MiSeq 250 and MiSeq 300-Cycle v2 Reagent Kit (Illumina).

Base calling was performed using the Illumina pipeline software. A total of approx 2.1 Gb of pair-end (450 insert size, 44 % GC, 31 dupl level) and approx 5.6 Gb of mate-pairs (4–5 kb inserts, 44 % GC, 21 dupl level) were produced. Post sequencing analysis of the raw data (demultiplexing, trimming, ambiguous bases removal) were carried out using Illumina provided software and a specific pipeline implemented in Python.

### Genome assembly and confirmation of the taxonomic classification of *P. griseofulvum*

The reads of the three available libraries were filtered (quality > 10 and minimal read length > 31) and SPAdes [55] was used with default values to assemble the *P. griseofulvum* genome. PG3 assembled genome was compared with existing sequences of phylogenetic markers from the same and related species in order to early detect any unexpected error of misidentification, as reported for other *Penicillium* species [19]. Fragments of beta-tubulin and calmodulin, as marker genes, were used to perform a blastn against the genome of PG3, after alignment with the available sequences using Muscle v 3.8 [56] (Additional file 1: Table S10). The alignments were then concatenated and trimmed with TrimAl v1.3 [57] to remove all the gaps, then the phylogenetic tree was reconstructed with PhyML v3.0 [58].

### Gene prediction

Augustus was used to predict the genes encoded in the genome [12]. Parameters used for the prediction were taken from *Aspergillus nidulans*, which has been successfully used in the annotation of other *Penicillium* genomes [18]. Only complete genes were included in the final prediction. Genes were annotated using InterProscan [59].

### Phylome and species tree reconstruction

#### Phylome reconstruction

A phylome, the complete collection of phylogenetic trees for each gene encoded in a genome, was reconstructed for *P. griseofulvum*. Fourteen other species were included in the phylome. These comprised the other sequenced *Penicillium* genomes (*P. chrysogenum, P. oxalicum, P. roqueforti, P. camemberti, P. expansum, P. digitatum* and *P. italicum*) and members of the *Aspergillus* and *Talaromyces* clades (Table 2). The phylome was reconstructed using a previously used automatic pipeline that follows the steps needed to reconstruct phylogenetic trees: homology search, multiple sequence alignment and phylogenetic reconstruction. The process is described in more detail in Huerta-Cepas *et al.* (2011) [60]. Briefly, for each gene encoded in *P. griseofulvum* we performed a Smith-Waterman search against the database formed by the above mentioned 15 proteomes. Results were filtered according to e-value (<1e^−05^) and continuous overlapping region over the query sequence (>0.5). The number of homologs accepted was limited to the first 150. Multiple sequence alignments were then reconstructed for each group of homologs, using three different programs: Muscle v 3.8 [57], MAFFT v6.712b [61], and kalign [62]. Alignments were then performed in forward and reverse direction (Head or Tails approach) [63], and the 6 resulting alignments were combined into a consensus alignment with M-COFFEE [64]. This combined alignment was then trimmed to remove poorly aligned regions using trimAl v1.3 [57] (consistency-score cut-off 0.1667, gap-score cut-off 0.9). Finally we reconstructed the phylogenetic tree. In order to do this, we first selected the evolutionary model that best fits the data. Neighbour Joining trees were reconstructed as implemented in BioNJ [65] using seven different models (JTT, LG, WAG, Blosum62, MtREV, VT and Dayhoff) and the likelihood for each topology was calculated using PhyML v3.0, allowing for branch length optimization [58,59]; the two models best fitting the data, according to the AIC criterion [66], were used to derive ML trees. Four rate categories were used and invariant positions were inferred from the data. Branch support was computed using aLRT (approximate likelihood ratio test). The phylome can be found in phylomeDB v4 (phyID: 368) [67].

**Table 2:** List of species used during phylome reconstruction. Sources include UniProt (http://www.uniprot.org/), NCBI (http://www.ncbi.nlm.nih.gov/), http://fungalgenomes.org/data/, and in-house sequencing projects. If NCBI did not provide a gene prediction at the time a genome was downloaded, the gene prediction was done using AUGUSTUS [12].

**TaxID**	**Species name**	**Source**
5057	*Aspergillus clavatus*	UniProt
5059	*Aspergillus flavus*	UniProt
5061	*Aspergillus niger*	UniProt
5075	*Penicillium camemberti*	NCBI + Augustus
5076	*Penicillium chrysogenum*	http://fungalgenomes.org/data/
5082	*Penicillium roqueforti*	NCBI + Augustus
27334	*Penicillium expansum*	Sequencing project [19]
36651	*Penicillium digitatum*	Sequencing project [18]
40296	*Penicillium italicum*	Sequencing project [19]
162425	*Emericella nidulans*	UniProt
330879	*Aspergillus fumigatus*	UniProt
441959	*Talaromyces stipitatus*	NCBI
441960	*Penicillium marneffei*	http://fungalgenomes.org/data/
933388	*Penicillium oxalicum*	NCBI
1495726	*Penicillium griseofulvum*	Sequenced in this paper

#### Species tree reconstruction

A species tree was reconstructed using a gene concatenation approach. Encoding genes of the *P. griseofulvum* genome containing only one orthologous in each of the other species of interest were selected. 2,134 genes fulfilled the requirements. Their alignments, as constructed in the phylome, were then concatenated to form a super alignment that contained 1,479,842 amino acid positions. RaxML [68] was then used to reconstruct the species tree using the PROTGAMMALG model. Rapid bootstrap implemented in RAxML was used to calculate branch support for the species tree.

### Prediction of secondary metabolism clusters

A collection of 159 SM clusters were used to look for homologous clusters in the *P. griseofulvum* genome following the method described in Ballester and collaborators [19]. Briefly, a blast search was performed for each cluster and results were analysed for gene order. We considered the cluster present if at least 50 % of a SM cluster had homologs in *P. griseofulvum* and those genes were situated together in the genome. At least three genes needed to be present for the cluster to be considered. No more than three genes could be found between homologous genes that belonged to the cluster.

### *In vitro* growth and secondary metabolites production

Fungal growth and production of patulin, griseofulvin and roquefortine C were determined *in vitro* by centrally inoculating 55 mm PDA plates with a 5 μL drop of a conidial suspension at 10^6^ spores/mL and incubating the plate at 24 °C in the dark. Colony diameter (mm) and SM production were recorded for up to 10 days. Patulin, griseofulvin and roquefortine C were extracted simultaneously from the mycelium and the medium by washing the Petri plates with 3 mL methanol. After 10 min incubation, the liquid was collected and centrifuged at room temperature and 10,000 × *g* for 5 min. Biomass was collected and stored at −80 °C until RNA extraction. The supernatant was filtered through a 0.45 μm syringe filter and analysed by HPLC. Results were expressed as μg of SM per plate. Chromatography was carried out with a Waters HPLC system equipped with a 600 quaternary pump and a 996 photodiode array detector. Data were analysed with the Empower software (Waters). Patulin, griseofulvin and roquefortine C were separated using a Luna C18 reverse column (250 x 4.6 mm, 5 μm; Phenomenex) coupled to a μBondapak C18 guard column (10 μm). Samples were separated at a flow rate of 1 mL/min on a linear gradient of 5 to 95 % solvent B in 30 min followed by isocratic 95 % solvent B for another 15 min (solvent A: acidic water pH 4.0 with acetic acid, solvent B; acetonitrile); the injection volume was 20 μL. The spectra were acquired between 200 nm and 600 nm wavelength, and patulin, griseofulvin and roquefortine C quantifications were performed at 274, 294 and 304 nm, respectively. Patulin, griseofulvin and roquefortine C were identified in the samples by comparing the retention time and UV–vis spectra with those of authentic standards.

### Analysis of SM production on apples

Patulin, griseofulvin and roquefortine C production by *P. griseofulvum* PG3 were quantified on apples (*Malus* x *domestica* cv. Golden Delicious) following the method described by Spadaro and collaborators (2013) [69] with some modifications.

First, conidial suspensions used for fruit inoculation were prepared by growing the pathogen in Petri dishes on PDA containing 25 mg/L streptomycin. After one week of incubation at 22 °C, conidia were collected.

Secondly, the fruits were disinfected and then punctured with a sterile needle at the equatorial region (3 mm depth/wide; 3 wounds per fruit).

Apples were inoculated with a spore suspension of *P. griseofulvum* PG3 (5 × 10^6^ con/mL), and then were stored at 22 ± 1 °C. At 8, 9 and 10 dpi (days post inoculation), patulin, griseofulvin and roquefortine C were extracted from blue mould rot tissue caused by *P. griseofulvum* following the method described below. Briefly, twenty grams of sample were placed in a centrifuge tube to which 20 drops of pectinase enzyme solution (Sigma Chemical Co., St Louis, USA; 5 U/g juice) and 10 mL water were added. The mixture was left at 38 °C for 2 hours and then centrifuged at 4,500 rpm for 5 min. Five mL clear juice were placed into a separating funnel; patulin, griseofulvin and roquefortine C were extracted with 15 mL ethyl acetate shaking for 1 min. The organic layer was separated from the water layer. The procedure was repeated three times. The sample was moved into a round bottom flask through a funnel and filter paper containing 25 g sodium sulphate anhydrous and evaporated to dryness (Rotavapor Laborota 4000, Heidolph, Schwaback, Germany). The residual was resuspended with 1 mL acidic water (pH 4.0 with acetic acid) and analysed in similar HPLC system as extracts of the pure cultures.

### RNA extraction and cDNA synthesis

Total RNA was extracted from 250 mg frozen mycelium according the protocol described by Ballester and collaborators [19]. Quality and concentration of total RNA was analysed by gel electrophoresis and spectrophotometer. DNase treatment and first-strand cDNA synthesis were conducted with the ‘Maxima H Minus First Strand cDNA Synthesis Kit with dsDNase’ (Thermo Scientific) using 2 μg of total RNA.

### Analysis of gene expression by RT-qPCR

RT-qPCR was performed following MIQE guidelines [70,71]. Gene-specific primer sets were designed for gene expression analysis (Additional file 1: Table S11) with Primer3Plus [71]. LightCycler480 System (Roche) was used. with SYBR Green to monitor cDNA amplification; cycling conditions were 5 min at 95 °C, followed by 45 cycles of 10 s at 95 °C, 5 s at 58 °C and 10 s at 72 °C. For each primer pair and each sample, the PCR efficiency (E) and the quantification cycle (Cq) were assessed using LinRegPCR software version 2014.2 [72]. Amplicon specificity was examined by analysis of the melting curve. Relative gene expression of the target gene was calculated based on the E and Cq values of the target and the reference genes according the following equation: E_target_^^(−Cqtarget)^ / E_ref_^^(−Cqref)^ [73]. The Cq value for the reference normalization factor was calculated by taking the geometric mean of the four *P. griseofulvum* reference genes: the ribosomal 28S RNA, the 37S ribosomal protein (PGRI_092740), the beta-tubulin (PGRI_052690), and the histone H3 (PGRI_044770)). Three independent biological replicates with at least two technical replicates were performed for each sample.

### Statistical analysis

All statistical analyses were performed with SPSS version 21.0 (SPSS Inc., Chicago, IL, USA). Data obtained in all the experiments were analyzed using analysis of variance (ANOVA). The treatment means were separated at the 5 % significance level by using Tukey's multiple range tests. Values are presented as the mean ± SD (standard deviation of the mean). The results are the mean of three independent experiments.

### Availability of supporting data

The data sets supporting the results of this article are included within the article and its additional files.

## Additional file

Additional file 1:
**Figure S1.** Colony diameter (A) and view (B) of *P. griseofulvum* strain PG3 (PGRI). Spore suspension of PG3 was inoculated on the centre of 55 mm PDA plates and incubated at 24 °C in the dark for up to 10 days post inoculation (dpi). Error bars indicate standard deviations of three biological replicates. **Figure S2.** Confirmation of the taxonomic classification of the newly sequenced genome of the *P. griseofulvum* PG3. PG3 sequence was compared with fragments of the beta-tubulin and calmodulin of different *Penicillium* species retrieved from GenBank. **Table S1.** SMURF comparison of NRPS, PKS, NRPS-PKS and DMAT between PG3 and the other sequenced *Penicillium* genomes. Total number of signature proteins include also the "PKS-Like" and "NRPS-Like" proteins. **Table S2.** List of SM clusters identified in *P. griseofulvum* PG3. **Table S3.** List of genes in the patulin gene cluster of *Penicillium griseofulvum* PG3 and putative function. **Table S4.** List of genes in the roquefortine C gene cluster of *Penicillium griseofulvum* PG3 and putative function. **Table S5.** List of genes in the griseofulvin gene cluster of *Penicillium griseofulvum* PG3 and putative function. **Table S6**. List of genes in the penicillin gene cluster of *Penicillium griseofulvum* PG3 and putative function. **Table S7. **List of genes in the cyclopiazonic acid gene cluster of *Penicillium griseofulvum* PG3 and putative function. **Table S8.** List of genes in the yanuthone D gene cluster of *Penicillium griseofulvum* PG3 and putative function. **Table S9.** List of genes in the chanoclavine I gene cluster of *Penicillium griseofulvum* PG3 and putative function. **Table S10.** Genbank accession numbers of the marker genes (beta-tubulin and calmodulin) used for the confirmation of the taxonomic classification of PG3. Table S11 List of primers used in this study. (ZIP 448 kb)

## Abbreviations

SM: Secondary Metabolites
PDA: Potato Dextrose Agar
SMURF: *Secondary Metabolite* Unique Regions Finder
NRPS: Nonribosomal Peptides
PKS: Polyketide Synthase
MIBiG: Minimum Information about a Biosynthetic Gene Cluster
Dpi: Day Post inoculation
MFS: The Major Facilitator Superfamily.
