# Supplementary material for: Genome sequencing and secondary metabolism of the postharvest pathogen Penicillium griseofulvum
Source: BMC Genomics. 2016 Jan 5;17:19. doi: 10.1186/s12864-015-2347-x (PMC4700700; doi:10.1186/s12864-015-2347-x)
Supplement: Additional file 1: — Figure S1. Colony diameter (A) and view (B) of P. griseofulvum strain PG3 (PGRI). Spore suspension of PG3 was inoculated on the centre of 55 mm PDA plates and incubated at 24 °C in the dark for up to 10 days post inoculation (dpi). Error bars indicate standard deviations of three biological replicates. Figure S2. Confirmation of the taxonomic classification of the newly sequenced genome of the P. griseofulvum PG3. PG3 sequence was compared with fragments of the beta-tubulin and calmodulin of different Penicillium species retrieved from GenBank. Table S1. SMURF comparison of NRPS, PKS, NRPS-PKS and DMAT between PG3 and the other sequenced Penicillium genomes. Total number of signature proteins include also the "PKS-Like" and "NRPS-Like" proteins. Table S2. List of SM clusters identified in P. griseofulvum PG3. Table S3. List of genes in the patulin gene cluster of Penicillium griseofulvum PG3 and putative function. Table S4. List of genes in the roquefortine C gene cluster of Penicillium griseofulvum PG3 and putative function. Table S5. List of genes in the griseofulvin gene cluster of Penicillium griseofulvum PG3 and putative function. Table S6. List of genes in the penicillin gene cluster of Penicillium griseofulvum PG3 and putative function. Table S7. List of genes in the cyclopiazonic acid gene cluster of Penicillium griseofulvum PG3 and putative function. Table S8. List of genes in the yanuthone D gene cluster of Penicillium griseofulvum PG3 and putative function. Table S9. List of genes in the chanoclavine I gene cluster of Penicillium griseofulvum PG3 and putative function. Table S10. Genbank accession numbers of the marker genes (beta-tubulin and calmodulin) used for the confirmation of the taxonomic classification of PG3. Table S11 List of primers used in this study. (ZIP 448 kb) [file 12864_2015_2347_MOESM1_ESM.zip › Table S8.docx]

**Table S8**

List of genes in the Yanuthone D gene cluster of *Penicillium griseofulvum* PG3 and putative function.

| **Secondary metabolite** | **Gene name** | **Gene code** | **Putative function** |
| --- | --- | --- | --- |
| Yanuthone D | *yanA* | PGRI_091150 | 6-MSA synthase |
| Yanuthone D | *yanB* | PGRI_091140 | 6-MSA decarboxylase |
| Yanuthone D | *yanC* | PGRI_091130 | m-Cresol hydroxylase |
| Yanuthone D | *yanD* | PGRI_091180 | Dehydrogenase |
| Yanuthone D | *yanE* | PGRI_091190 | Oxidase |
| Yanuthone D | *yanF* | PGRI_091210 | Uncharacterized protein |
| Yanuthone D | *yanG* | PGRI_091140 | Prenyltransferase |
| Yanuthone D | *yanH* | PGRI_091160 | Cytochrome P450 |
| Yanuthone D | *yanI* | PGRI_091170 | O-Mevalon transferase |
| Yanuthone D | *yanR* | PGRI_091120 | Transcription factor |
